# Factors associated with compliance with weekly iron and folic acid supplementation among school adolescent girls in Debub Achefer district, northwest Ethiopia: school-based cross-sectional study

**DOI:** 10.1038/s41598-024-60800-5

**Published:** 2024-05-01

**Authors:** Bisrat Haile, Abdu Oumer, Tarkegn Negese, Mesfin Temesgen, Aweke Kebede, Dureti Abdurahman, Aboma Motuma, Kedir Teji Roba

**Affiliations:** 1 Nutrition Coordination Office, Ministry of Health, Addis Ababa, Ethiopia; 2 College of Health and Medical Sciences, Haramaya University, Harar, Ethiopia; 3 College of Health and Medical Sciences, Bahir Dar University, Bahir Dar, Ethiopia; 4 World Food Program, Addis Ababa, Ethiopia

**Keywords:** Adolescent girls, Compliance, Northern Ethiopia, Supplementation, Weekly IFAS, Health policy, Nutrition, Public health

## Abstract

Iron deficiency anemia is a public health problem among adolescents that could be addressed by weekly Iron Folic Acid Supplementation (IFAS). The Ethiopian government piloted weekly IFAS in schools, where its effectiveness depends on compliance. We assessed the determinants of compliance with the weekly IFAS in Ethiopia. A school-based survey was conducted in 506 adolescent girls on weekly IFAS. Compliance was considered when girls reported WIFAS for at least three months without discontinuation. Bivariable and multivariable logistic regression models were modeled, with odds ratios reported. Out of 506, 25.8% had limited access to educational resources, and 79.4% had no information on IFAS. Among these, 47.9% (95% CI: 45.5–49.9%) had poor compliance with weekly IFAS. Non-compliance was mainly due to school absenteeism (55.9%). Important predictors of poor compliance were adolescent girls’ marital status (AOR = 5.21; 1.55–17.6), academic standing (AOR = 4.37; 2.20–8.70), family income (AOR = 1.85; 1.09–3.15), access to health education materials (AOR = 1.57; 1.02–2.40), problems with IFAS (AOR = 2.44; 1.26–4.74), a discouraging home environment for the program (AOR = 2.27; 1.54–3.34), and a lack of knowledge of the IFAS program (AOR = 1.40; 0.97–2.03). Compliance with weekly IFAS is optimal, which could be improved via strong adherence support and feasible supplementation schedules.

## Introduction

Nutrition has a profound impact on the current and future health of adolescents, contributing to a reduced burden of non-communicable diseases in adulthood^1^. Hence, investing in adolescent nutrition helps to achieve adolescents’ health, improved well-being, productivity, and reduced health risks for their children^2,3^. Among these, iron has a profound effect on adolescent health and is essential for promoting growth and development, metabolism, and the production of hemoglobin to prevent anemia^4,5^.

Iron deficiency is the leading cause of anemia in developing countries, accounting for around 40% of all cases^4,6^. Anemia affects more than 577.9 million (32.5%) adolescent girls and women worldwide, with Sub-Saharan Africa (SSA) bearing the most burden^7^. Adolescent girls are more vulnerable to iron deficiency anemia (IDA) due to lower iron intake and frequent menstrual cycles. It is linked to poor academic achievement, low productivity, stunted growth and development, and poor maternity outcomes^4,8^. Adolescent girls are thus a high-risk population group for anemia and its negative implications^5,7,9^. When adolescents become pregnant, their chances of having a low birth weight, a stillbirth, or a preterm birth increase^10^. According to the Ethiopia demographic and health census, 24% of women of reproductive age in Ethiopia were anemic^11^. According to study conducted in Ethiopia, 27–45% of adolescents were anemic, indicating that anemia is a moderate public health problem among adolescent girls^12–14^.

Among the different techniques for treating anemia, daily or intermittent iron supplementation for high-risk groups is broadly acknowledged^8,15^, depending on the epidemiology of anemia. In locations with moderate to severe public health problems (prevalence greater than 40%), daily supplementation is indicated, whereas intermittent supplementation is recommended in mild to moderate situations^16^. Studies indicate that weekly or intermittent iron and folic acid supplementation (IFAS) results in higher bioavailability and compliance^15,17–19^. Similar to World Health Organization (WHO)^16^, the national micronutrient recommendation suggests weekly IFAS for adolescent girls (10–19 years) (60 mg elemental iron and 2.8 mg folic acid) as an effective strategy to address anemia^20^.

Intermittent IFAS is significant due to its operational feasibility and effectiveness, particularly for school-age adolescents. However, supplement compliance is critical for program effectiveness, and numerous studies have found poor compliance^18,19,21–23^.

Adolescent adherence to IFAS is exceedingly low in certain African countries, particularly in places where school attendance is inconsistent^24^. Studies showed that 46–55.3% of women had good adherence to IFA^25,26^ indicating a poor adherence, affecting the supplementation effectiveness. Furthermore, there is paucity of evidence based practical implementation of weekly IFAS in the area and the nation, where such evidence could support existing initiatives. As a result, the current study was designed to measure compliance and its determinants for weekly IFAS among these adolescents in the selected group. As a result, the findings of this study provide evidence-based information to programmers and stakeholders in deciding on the subnational and national scale-up of weekly IFAS, as well as information on the school-base modality of weekly IFAS.

## Results

### Socio-demographic characteristics

Among 543 school-age adolescent girls, 506 of them were on IFAS and included in the current study. Regarding the age of the adolescent girls, 67.6 and 32.4% of them were aged 10–14 and 15–19, respectively. The majority (97%) of them were not married, and 68% were attending in grade 5–8. Farming was the main source of income for 83.1% of their families, followed by merchants (11.8%). Moreover, a total of 80.5% of adolescents reported the availability drinking waters in schools (Table 1).

**Table 1:** Socio-demographic characteristics of school-age girls on weekly IFAS in Debub Achefer district, northern Ethiopia, 2019.

Variables	Category	Frequency	%
Age in years	10–14	367	67.6
	15–19	176	32.4
Marital status	Single	527	97.1
	Married	16	2.9
Grade attained	1–4	78	14.3
	5–8	369	68.0
	9–10	96	17.7
Family source of income (occupation)	Farmer	451	83.0
	Government employee	17	3.1
	Private employee	8	1.5
	Daily laborer	3	0.6
	Merchant	64	11.8
Availability of drinking water in schools	Yes	437	80.5
	No	106	19.5

### Knowledge and utilization of weekly IFAS services

About 76.8% adolescent girls responded that the IFA tablet should be taken for four weeks. More than two-third (38.8%) of them responded that taking IFA protects against anemia. Moreover, 72.7% of adolescent girls were aware that adolescence is a critical period for nutrition, whereas only 55.8% of them reported that they had ever heard about weekly IFAS. Of the total adolescent girls, only 25.8% of them accessed any type of social behavioral change communication (SBCC) materials related to weekly IFAS. The main sources of information for students were teachers (85%), relatives (8.9%), and girl clubs and media (13; 4.3%) regarding weekly IFAS in the school. Regarding their knowledge score, a 70-percentile cutoff point was used to classify good and poor knowledge on the importance of IFAS (Table 2).

**Table 2:** Knowledge of adolescent girls on weekly IFAS at Debub Achefer district, Amhara region, northern Ethiopia, 2019.

Question	Category	Frequency	Percent (%)
For how long is IFA tablet should be taken within a month?	For one week	41	7.6
	For three weeks	57	10.4
	For three weeks	28	5.2
	For four weeks and above	417	76
How many tablets should be taken within a year?	12 tablets	57	10.5
	24 tablets	47	8.6
	No limit	8	1.5
	I don’t know	431	79.4
What is the appropriate age to start taking weekly IFA for adolescent girls?	At 10 years of age	207	38.1
	At 15 years of age	69	12.7
	At 18 years of age	13	2.4
	Don’t know	254	46.8
What is the importance of taking weekly IFAS	For good school performance	52	8.7
	Protection against anemia	231	38.8
	I don’t know	85	14.3
	Other health benefits	227	38.2

### Compliance to weekly IFAS

This study found that 93.2% adolescent girls were taking IFA at the time of data collection. Almost one-fifth (19.2%) of the participants respond that they stopped using IFA because of side effects, family permission, absence during supplementation, or other potential personal misconceptions among those who were already taking IFA. Only 66.1% (95% CI: 64–68.2%) of them had been taking IFA for three months or more, indicating strong compliance with the weekly IFAS Program (Table 3).

**Table 3:** Level of awareness of IFAS and utilization patterns of weekly IFAS among adolescent girls from the selected schools in Debub Achefer district, northern Ethiopia, 2019.

Items	Category	Frequency (n)	Percent (%)
Are you aware of that Adolescence is a critical period for nutrition	No	148	27.3
	Yes	395	72.7
Have you ever heard about Weekly IFAS?	No	240	44.2
	Yes	303	55.8
Have you ever accessed any type of SBCC material related to IFAS?	No	403	74.2
	Yes	140	25.8
Are you taking IFA currently?	No	37	6.8
	Yes	506	93.2
For how long you have been taking	Less than three months	184	33.9
	For three months or more	359	66.1
The reason for not taking IFA	Fear of side effect	2	5.4
	No permission from parents	4	10.8
	No information on the program	14	37.8
	Other^a^	17	45.9
Is there a time you interrupted taking your weekly IFA?	No	402	74.0
	Yes	104	19.2
The reasons for IFA interruption	Side effect	13	12.3
	Absent from school	59	55.7
	No IFA supply	4	3.8
	Others^b^	30	28.3

a, refers to due to age restriction, misconception with the IFAS will be contraception and might cause infertility and b, indicates sickness, menstruation, taking other medications and misconceptions with IFAS tablets.

Based on the three reported IFAS experiences, we built compliance for weekly IFAS. Thus, while 52.1 (95% CI: 50.0–54.2% of adolescent girls had good compliance, 260 (47.9%; 95% CI: 45.5–49.9%) exhibited low compliance to weekly IFAS (Table 3). Among the respondents, 93.2% were taking IFA at the time of data collection. In disaggregated forms, while 19.2% of them have interrupted taking IFA due to various reasons, 6.1% (95% CI: 64–68.2%) of them have been taking IFA for three months and above. The main reported reason for interruption of IFAS was school absenteeism (55.9%) during the time of supplementation (IFAS), and 12.3% were due to IFAS-related side effects (Table 3).

### Facilitators or barriers to compliance to weekly IFAS

About 67.4% of the adolescent girls in the study had discussed their decision to take IFA with their families. Of which, 61.7% of those who spoke with a family member received a positive response. Almost one-third of them did not consult or discuss the matter with their family members. Surprisingly, 90.6% of adolescent girls reported no negative effects after using IFA on a weekly basis (Table 4).

**Table 4:** Facilitators or barriers to weekly IFAS among adolescent girls from the selected schools in Debub Achefer district, northern Ethiopia, 2019.

Questions	Category	Frequency	Percent
Have you discussed with your family on your status of taking IFA?	No	177	32.6
	Yes	366	67.4
What was your family members’ response during discussion of IFA taking?	Encouraging	335	61.7
	Discouraging	19	3.5
	No response	12	2.2
To whom you discussed the issue?	Teachers	339	61.0
	Girls club	17	3.1
	Media	2	0.4
	Relatives	199	35.8
Have you ever encountered any form of trouble after you have taken your IFA tablet?	No	492	90.6
	Yes	51	9.4
What kind of problem you encountered?	Heart burn	21	41.2
	Increased in menstrual flow	9	17.6
	Others^a^	23	43.4
What measures you have taken?	Went to health facility for advice	11	21.6
	Temporary discontinued taking the tablet	8	15.7
	No measure was taken	32	62.7
What do you think are the reasons for Adolescent girls for not taking or discontinuing weekly IFA?	lack of information	162	39.7
	Family decision	140	34.3
	Fear of adverse effect	167	40.9
	Others^b^	89	21.8

a, includes abdominal pain, headache, chills, and vomiting; b, includes absent from school, age limit, and other misconceptions.

### Factors associated with compliance to weekly IFAS

We run separate bivariable and multivariable (adjusted model) regressions to identify possible risk factors for poor compliance with weekly IFAS. All the proposed variables were run using cross-tabulation, and only those relevant or significant contributors were included in the final adjusted model. In the non-adjusted model, married adolescent girls, attending grades 1–4, girls from public and private schools, those who had experienced some problems (side effects) with IFAS, a discouraging home environment, and poor knowledge of IFAS were important determinants of compliance with weekly IFAS.

In multivariable regression, girls who were married (AOR = 5.21; 95% CI: 1.55–17.6), low-grade 1–4 (AOR = 4.37; 95% CI: 2.20–8.70), and from non–government working families (AOR = 1.85; 95% CI: 1.09–3.15)) were positively associated with poor-compliance with the weekly IFAS. The odds of poor compliance were significantly higher among those adolescent girls without access to health education materials (AOR = 1.57; 95% CI: 1.02–2.40) and who encountered some problems with IFAS (AOR = 2.44; 95% CI: 1.26–4.74) as compared to their counterparts. The odds of having poor compliance with IFA in the school were higher among those without discussion with family members than among those who had discussion with family members. Moreover, discouraging home environments for the IFAS program (AOR = 2.27; 95% CI: 1.54–3.34) and having poor knowledge of the IFAS program (AOR = 1.40; 95% CI: 0.97–2.03) were associated with higher odds of poor compliance with the weekly IFAS (Table 5).

**Table 5:** Factors associated with compliance to weekly IFAS at Debub Achefer district, Amhara Region, northern Ethiopia, 2019.

Factors	Codes	Compliance to weekly IFAS		COR with 95% CI	AOR (95% C.I)	*P*-value
		Poor compliance	Good compliance			
Age in years	10–14	186 (50.7%)	181 (49.3%)	1		
	15–19	74 (42%)	102 (58%)	1.42 (0.99–2.04)		
Marital status	Single	248 (47.1%)	279 (52.9%)	1	1	
	Married	12 (75%)	4 (25%)	3.38 1.08–10.6)*	5.21 (1.55–17.6)*	0.008
Family source of income (occupation)	Farmer	209 (46.3%)	242 (53.7%)	1.23 (0.46–3.30)		
	Private	44 (58.7%)	31 (41.3%)	2.03 (0.70–5.91)		
	Government	7 (41.2%)	10 (58.8%)	1		
Grade	Grade 1–4	51 (65.4%)	27 (34.6%)	3.16 (1.69–5.87)**	4.37 (2.20–8.70)**	0.0001
	Grade 5–8	173 (46.9%)	196 (53.1%)	1.47 (0.93–2.33)	2.00 (1.18–3.38)*	0.010
	Grade 9–10	36 (37.5%)	60 (62.5%)	1	1	
Occupation	Farmer	209 (46.3%)	242 (53.7%)	1.23 (0.46–3.30)	1.85 (1.09–3.15)*	0.024
	Private	44 (58.7%)	31 (41.3%)	2.03 (0.70–5.91)	1.55 (0.60–4.31)	0.399
	Government	7 (41.2%)	10 (58.8%)	1	1	
Access to any SBCC material	No	206 (51.1%)	197 (48.9%)	1.67 (1.13–2.47)*	1.57 (1.02–2.40)*	0.039
	Yes	54 (38.6%)	86 (61.4%)	1	1	
Side effects	No	230 (46.7%)	262 (53.3%)	1	1	
	Yes	30 (58.8%)	21 (41.2%)	1.63 (0.91–2.92)	2.44 (1.26–4.74)*	0.008
Availability of drinking water	No	62 (58.5%)	44 (41.5%)	1.70 (1.11–2.61)*		
	Yes	198 (45.3%)	239 (54.7%)			
Decision	No	238 (47.3%)	265 (52.7%)	1		
	Yes	22 (55%)	18 (45%)	1.36 (0.71–2.60		
Discussion with family on IFAS	No	101 (57.1%)	76 (42.9%)	1.73 (1.20–2.49)**		
	Yes	159 (43.4%)	207 (56.6%)	1		
Home environment for IFAS	Encouraging	138 (41.2%)	197 (58.8%)	1	1	
	Discouraging	122 (58.7%)	86 (41.3%)	2.03 (1.43–2.88)**	2.27 (1.54–3.34)**	0.0001
Knowledge on IFAS	Good knowledge	127 (41.8%)	177 (58.2%)	1	1	
	Poor knowledge	133 (55.6%)	106 (44.4%)	1.75 (1.24–2.46)**	1.40 (0.97–2.03)	0.073

Statistically significant covariates of compliance to weekly IFAS at *p*-value below 0.05 (*) and 0.001(**).

## Discussion

The current study was to investigate the level of compliance with weekly IFAS and important factors affecting compliance among adolescent girls in Debub Achefer district, Ethiopia, where national weekly IFAS was piloted. According to this study, only 66.1% of adolescent girls had good compliance with IFAS which indicates a promising compliance yet to be improved. The national guideline recommends adolescent girls to take at least twelve tablets of IFA within three months for better effectiveness with universal compliance^20^. However, the higher noncompliance (33.9%) was reported in the current study, which could limit its effectiveness in reducing the burden of anemia. On the contrary, the current compliance rate is very similar to studies reported from pilot conducted in Ethiopia, where 62.9% of them consumed the full dose (24 tablets) during the six-months period^22^. On the other hand, the current non-compliance rate is lower than studies conducted at Bahour commune in rural Puducherry public schools (47.2%)^27^, and rural Pondicherry (85.8%)^28^.

Additionally, 93.2% of adolescent girls have taken IFA at least once in the first semester of the academic year. This experience agrees with the technical report of operational research conducted by AAU that showed 89.3 and 73.8% were on the weekly IFAS program^22^. This high percentage of adolescent girls who ever started taking IFA in the aforementioned study could be due to collaborative efforts of stakeholders involved in the program. It is also obvious that activities like training, supply provision, and close follow up at the beginning of a new program may result in a high utilization rate^20^. But if the same effort is maintained throughout the program implementation, persistently higher compliance and better outcomes could be achieved.

Related to this, one-fifth of girls interrupted IFA, mainly due to school absences, side effects, or a lack of IFA supply. In different studies, the reasons for interrupting IFA were the occurrence of side effects;- nausea or vomiting, bad taste; misconceptions, and being absent from school on the date of supplementation^18,19,29,30^. Also, thirty seven girls did not completely accept IFA, which was mainly linked to a lack of clear information and misconceptions about the IFA tablets. This implies the need for enhanced community engagement and BCC intervention to increase adoption and address context-specific misconceptions^3,28,31^. Scaling weekly IFAS schedules usually requires strong collaborative work by creating community engagement and making adolescents understand the temporary nature of these incidents.

The study shows that those girls who had experiencing common side effects on weekly IFAS had lower compliance to IFAS. Although the common side effects of IFAS-heart burn, nausea, metallic taste, and discomfort are common, the weekly administration could significantly reduce their occurrence and improve compliance^32^. However, side effects may terrify girls and testify to some misconceptions in the community. It is important to have a strong behavioral change intervention focusing on the transitory nature of these side effects^15,33^.

In the current study, adolescent girls who were married and being from nongovernmental worker families were more likely to have poor compliance with weekly IFAS compared to their counterparts. These could be related to a better home environment and knowledge on the issue among girls among governmental workers as compared to farmers and private workers. Hence, it could be a result of better education and a favorable attitude toward health issues. However, unlike the findings of this study, another study indicated that there is an association between compliance with IFA supplementation and increasing age^19^ that could also be associated with the grade level.

The study also showed that the presence of a discouraging at home environment and poor knowledge of IFAS were associated with noncompliance among adolescent girls. For instance, a study indicated that modeled behavioral interventions could improve mean knowledge scores, attitude, subjective norm, perceived behavioral control, and behavioral intention for positive behaviors^34^. Therefore, the IFAS program should establish functional BCC and community engagement packages through the use of influential local leaders to improve the home environment for better adoption. This would increase their awareness and knowledge and help their family understand the advantages of compliance with IFA tablets. It should be noted that public hesitance usually exists in the first few months of the program and might get better thereafter^32^.

Adolescent girls with limited access to SBCC material were 57% higher odds of noncompliance with weekly IFAS compared with their counterparts. Thus, poor access to educational materials could limit their exposure to information about the IFAS program and make them absent from school during supplementation schedules. In addition, access to BCC materials further promotes open discussion with family members for better compliance. For example, a study showed that noncompliance and supplement interruption were related to parental recommendations^21^.

### Limitations of the study

The results from this study were self-reported by the adolescent girls, so there is a possible tendency to recall bias, and girls may tend to overestimate their compliance. However, we assessed compliance using three separate yet interconnected questions to capture the real experience and cross-validate their response. This could reduce haphazard responses to compliance with IFAS.

## Conclusion

Compliance with the weekly IFAS was found to be promising yet to be enhanced via various targeted behavioral change interventions and better program designs. Also, non-compliance to weekly IFAS was found to be associated with poor knowledge, access to SBCC materials, a discouraging home environment, awareness, and school absenteeism. This can further be improved by increasing access to health education materials, flexible supplementation schedules, and the participatory involvement of stakeholders. Strong adherence support and feasible supplementation schedules could help increase compliance with the weekly IFAS. Hence, it would be very helpful to increase access to educational materials and deliver health education to increase compliance with supplementation. The findings of this study could implicate things to foster while scaling this program at a larger scale.

## Materials and methods

### Study setting, and intervention settings and modality

The school-based cross-sectional study was done in randomly selected schools in northern Ethiopia’s Debub Achefer district, West Gojam Zone, in Amhara Regional State. The district is located at an average elevation of 2300 m with an annual temperature of 16.7–37.6 °C. According to the Ethiopian Statistical Service’s population projection for 2022, the district had a total population of 176,088 people, with 88,990 men and 87,098 women, in a total area of 1102 square kilometers^35^. The district has diverse agricultural practices, mainly mixed cropping and animal keeping. Maize is the main staple crop produced and consumed in the area. A study showed that about 11% of pregnant women had anemia^36^. Moreover, there is evidence of prevailing intestinal parasitosis and it affects causes anemia, which is 37% of pregnant women (hookworm, 18.2%)^37^. About 32 and 11% of school children were undernourished and anemic, respectively^38^, indicting the prevailing malnutrition in the study area. The district has 35 schools that started the weekly IFAS program at the time of this study. According to the national recommendation, 6668 of the 14,833 adolescent girls in the area were taking the IFA supplement per week at these schools (Program Report, Achefer 2018/19). The survey took place throughout the first three weeks of January 2019.

The current study focuses on adolescents who participate in a weekly IFAS and nutrition education program that is largely delivered by National Nutrition Program (NNP II) implementing sectors and their institutions. These interventions were implemented in selected schools, primary health care units (PHCUs), and community structures such as the Health Development Army in the districts chosen for this initiative. The service was provided using the school’s weekly IFAS platform in collaboration with the district health and education office, the health facility’s integrated outreach platform, and the health facility’s routine weekly IFAS and nutrition education delivery platform.

The previous service was provided in the form of an observed weekly IFAS or taking the supplement at home. However, the current intervention employed an integrated supplementation and behavioral change intervention through nutrition education delivered by service providers. The interventions were delivered as pilot government initiative by trained staffs, and school teachers along with targeted behavioral interventions. The NNP II was in charge of carrying out this program. The current study targeted only adolescent girls attending school who have been taking weekly IFAS; they were included for the sake of physical accessibility and a large share of the program.

### Study participants

This study primarily targeted all adolescent girls aged 10–19 who receive weekly IFAS at schools in the Debub Achefer district of northern Ethiopia. Then, a random sample of schools and adolescent females from each school were assessed to determine compliance and its determinants for weekly IFAS at the study location. Hence, all 506 adolescent girls who were taking weekly IFAS from the chosen schools, were included in the study.

### Sample size and sampling procedures

The sample size was estimated using the single population proportion formula, assuming a prevalence of compliance (66%)^22^, 5% level of significance at 95% confidence level, 5% non-response rate, and a design effect of 1.5 to account for possible clustering and heterogeneity in the outcome measured. A marginal error of 5% around the point prevalence of compliance rate was assumed where a total of 543 adolescents were required for this study. Among these a total of 506 adolescent girls were actually included in the study.

The district was carefully chosen because it is one of the Ministry of Health’s experimental districts for the weekly IFAS intervention area. Six schools were chosen at random from a total of 35 that were targeted for this intervention. Then, the sample was proportionally allocated to each randomly selected schools where adolescents girls were randomly selected for interview. As a consequence, 62 adolescents (from Aferida school), 88 (from Nifasa school), 65 (from Korench school), 99 (from Lalibela school), 135 (from Ashuda school), and 94 (from Lihudi school) were picked for this study.

### Data collection procedures and quality assurance

The data was obtained using a standardized, pretested, and translated questionnaire via the Open Data Kit (ODK) offline data collection tool. The questionnaire was created using previous material and in Amharic, the local language. A team of skilled and experienced data collectors was assembled to conduct face-to-face interviews with adolescent girls. Compliance was measured through self-reported absences from the weekly scheduled IFAS tablets. This method is more valid and less prone to recollection bias and respondent burden because the tablets are normally given weekly for around three months as opposed to daily doses. This would suggest complete program compliance, but noncompliance could lead to IFAS’s ineffectiveness. Hence, compliance to weekly IFAS was defined as taking or not missing any weekly schedules for consecutive three months. If a girl takes a consecutive 12 tablets over the three month period without interruption, she is classified as compliant to weekly IFAS.

Six trained data collectors with university degrees and extensive experience in data gathering techniques and field activities were hired. Data collectors and supervisors received one day of training on the tools, primarily the manner to capture data and the ODK system to capture data. A week before beginning data collecting in the selected schools, a pre-test of the questionnaire was completed on 10% of the samples. Throughout this data collection, the principal investigator (the first author) ensured that all data collection methods were followed.

### Variables in the study

The explanatory variables considered in this study were socio-demographic and socio-economic factors, knowledge of adolescents and adolescents’ attitudes toward weekly IFAS, availability of water in the school, sources of information, support from family and teachers, peer support, and other program-related factors. This study’s outcome variable was adolescent girl compliance with weekly IFAS, which was divided into two categories: good compliance and not complaining about the supplementation regimen. Weekly IFAS was defined as providing 60 mg of elemental iron and 2.8 mg of folic acid to teenage girls aged 10–19 years for three months. As a result, adolescent girls were deemed to be in good compliance with weekly IFAS if they took the supplement weekly for at least three months in the first semester of the academic year; otherwise, they were regarded as non-compliant^20–22^.

### Data processing and analysis

The data gathered using ODK was cleaned and reviewed for completeness by the data collectors and the supervisor on a daily basis before being sent to the server. SPSS 20 and STATA 14 were used for the analysis. The information was presented in the form of numbers, tables, graphs, medians, and means, as well as a measure of dispersion. Logistic regression assumptions were validated, and binary logistic regression analysis was utilized to discover characteristics associated with IFAS compliance in teenage girls. A bivariable binary logistic regression analysis was carried out and the results were given in the form of crude odds ratios and 95% confidence intervals. The adjusted models took into account variables with a statistically significant level of *p*-values below 0.25 on bivariable analysis and important potential determinants. To control for potential confounders, a stepwise backward regression approach was used, and an adjusted odds ratio and *p*-value were provided. The level of significance was fixed at *p*-values less than 0.05. Multicollinearity was checked using the Variance Inflation factor (VIF) test. The fitness of the model was assessed using Hosmer–Lemeshow’s goodness of fit at *p*-values greater than 0.05^39^. Potential interactions were also investigated, but none of the factors had statistically significant effect modification^39,40^.

### Ethical approvals

We received ethical permission from the Institutional Review Board of Bahir Dar University in Ethiopia for this research. Because this study included teenagers under the age of 18, both assent from the adolescent girls and informed consent from their parents, care givers, or legal guardians were obtained. The interview was held in a private room at the school that had been chosen in consultation with the school and the weekly IFAS coordinators. Personal and sensitive information were not recorded. For those who reported missing one or more IFAS tablets, systematic nutritional counseling focused on the importance, safety, dose, and need for the IFAS was provided for approximately 10 min. Meanwhile, doubts and misconceptions were cleared up in order to increase supplement adherence.

### Ethical approval and consent to participate

Formal ethical approval was obtained from Institutional Review Board of Bahir Dar University, Ethiopia. Both informed assent from adolescent girls and informed consent from their parents/caregivers or legal guardians were obtained. All methods and procedures were conducted in accordance with the approved ethical standard and with respect to studies involving human subjects in accordance with the Helsinki declaration.

## Data Availability

All the data generated in this study are within the submitted manuscript. Further datasets can be shared by corresponding author upon reasonable requests.

## Abbreviations

A/COR: Adjusted or crude odds ratio
AU: Addis Ababa university
BCC: Behavioral change communication
CI: Confidence interval
ECSC–SUN: Ethiopia civil society coalition–scale up nutrition
EDHS: Ethiopian demographic and health survey
HC: Health center
IFAS: Iron and folic acid supplementation
IFA: Iron and folic acid
MOH: Ministry of health
NI: Nutrition international
NNP: National nutrition program
ODK: Open data kit
PHCU: Primary health care unit
SBCC: Social and behavioral change communication
SSA: Sub Saharan Africa
WHO: World health organization
